# Nursing and medical students’ views on their knowledge related to the Sustainable Development Goals – a mixed methods study at three Swedish universities

**DOI:** 10.1186/s12909-025-06991-5

**Published:** 2025-03-25

**Authors:** Maria Niemi, Helle Mölsted Alvesson, Daniel Helldén, Olivia Biermann, Eva Henje, Helena Nordenstedt, Carl Johan Sundberg, Tobias Alfvén

**Affiliations:** 1 Department of Global Public Health, Karolinska Insititutet, Stockholm, Sweden; 2 Department of Clinical Sciences, Section of Child and Adolescent Psychiatry, Umeå University, Umeå, Sweden; 3 Department of Learning, Informatics, Management and Ethics, Karolinska Institutet, Stockholm, Sweden; 4 Department of Internal Medicine, Danderyd University Hospital, Stockholm, Sweden; 5 Department of Physiology and Pharmacology Stockholm, Karolinska Institutet, Stockholm, Sweden

**Keywords:** Sustainable development, Higher education, Medical professionals education, Students

## Abstract

**Background:**

The challenges that the world faces to ensure good life for future generations are vast and complex. The United Nations Sustainable Development Goals (SDGs) aim to meet these challenges. A growing number of higher education institutions have integrated them within their curricula, but there are indications that health professional education has been lagging behind. Therefore, it is important to better understand the views of students in health professional education on the level and depth of their education on sustainable development.

**Methods:**

This sequential exploratory mixed methods study was based on survey responses from *N* = 294 nursing (*N* = 137) and medical (*N* = 157) students of first and last semesters from three Swedish universities. From the full group of survey responders, 21 students participated in 5 focus group discussions (FGDs) and 9 individual interviews. The survey findings were summarized through descriptive statistics and the interviews and FGDs were analyzed by qualitative content analysis.

**Results:**

The survey findings showed that most students (63%) perceived that they had not learned enough about the SDGs and Agenda 2030 during their education, or for the purposes of their future career. Most of the students (63%) also thought that Agenda 2030 and the SDGs should be a greater part of their education. The qualitative data gave a more in-depth understanding of the quantitative findings, forming two themes: The first theme revealed that the SDGs may be more relevant for health care practice than what the students initially thought, but that the education they had received was in most places superficial, or not tied to the SDGs. The second theme detailed what and how students wished to learn more about. Here, they called for a more in-depth understanding of how to promote equality, equity, inclusion and psychosocial aspects in health care. They also hoped for more knowledge about how to ensure a sustainable working life for themselves.

**Conclusions:**

Nursing and medical students at three Swedish universities experience that they lack the knowledge necessary to face sustainability challenges they encounter in working life and give some suggestions about how this may be improved in future education.

**Supplementary Information:**

The online version contains supplementary material available at 10.1186/s12909-025-06991-5.

## Introduction

The challenges that the world faces to ensure good life on earth for current and future generations are vast and complex [1], and the UN's Sustainable Development Goals (SDGs) and Agenda 2030 aim to meet these challenges [2]. Ending poverty, improving health, social, and economic inequalities, improving education, and safeguarding a healthy environment are some of the SDGs [2]. Higher Education Institutions (HEIs) play an important role in contributing to these goals [3]. In particular, health professions education plays a role in equipping future health professionals with the competences needed to sustainably promote health and well-being, while also taking sustainable health into account [4]. Sustainable health has been defined as *“a multisectoral area for study, research, and practice towards improving health and well-being for all while staying within planetary boundaries*” [5]. Moreover, higher education should prepare future health professionals to address unresolved and complex social, political, economic and environmental challenges [1].

A Lancet Commission for educating health professionals in the new century [6] has highlighted that medical schools are ill-equipped to prepare students for the complexities they face in working life. The complex challenges of new infectious, environmental, and behavioral risks, as well as rapid demographic and epidemiological transitions, call for new forms of education that can help develop a more relational view of the world [7]. This is a challenge for health professions education, which is still mainly based on traditional medical disciplines, with a strong bias towards cognition, one-way knowledge transmission, and a reductionistic biomedical paradigm [8]. According to some scholars in education for sustainable development, a new form of education is thus called for, which should enable students to become more adept in capabilities like systems thinking, handling uncertainty, changing perspectives, moral reasoning, tapping into diversity, and actively engaging in change and transformation [9, 10]. Moreover, the notion of "students as change agents" has been highlighted as an important focus for medical education [6].

A growing number of HEIs have already integrated sustainable development within their curricula, research, operations, outreach, assessment and reporting [1]. The SDGs can be integrated into higher education across disciplines and in various ways, e.g., through mandatory or elective courses, workshops and lectures [11]. However, there is still a need for an in-depth understanding of the views of health professional students on the education for sustainable development they obtain during their degree programs, as students have been highlighted as important change agents for sustainable development [12–15] and a recent international survey study highlighted that planetary health is poorly incorporated into medical school curricula [16]. Moreover, the authors of a recent scoping review [17] recommend that future research and education development should focus on how to best integrate planetary health medical education. To address these gaps in knowledge, the aim of this study was to explore how medical and nursing students at three Swedish universities experienced their education for sustainable development which was offered as part of their study program and its potential relevance for their future working life. According to the Swedish Higher Educated Act, paragraph 5, “In their operations, higher education institutions shall promote sustainable development, which means that current and future generations are assured of a healthy and good environment, economic and social welfare and justice.” [18]. However, to our knowledge the integration of this mandate into health professions education has not yet been studied in the Swedish context.

## Methods

### Study design

This was a sequential exploratory mixed methods study [19] using a survey, focus groups and individual interviews to collect information about student experiences and views of students in health professional education on the level and depth of their education on sustainable development. The study was approved by the Stockholm Regional Ethical Review Authority with approval number 2021–04960, and complies with the Declaration of Helsinki in its latest version.

### Sampling

The inclusion criteria for the study were: Any student of the nursing or medical programs at either the first or final semester of their program respectively (semester six for nursing students, semester eleven for medical students) at Karolinska Institutet (KI), Uppsala University (UU) or Umeå University (UMU) in Sweden. Three different universities were chosen to allow for a broader perspective of how sustainable development is taught and learnt/experienced within nursing and medical education in Sweden.

### Recruitment

To recruit students to the study, names and e-mail lists of all students filling the inclusion criteria were requested from the study directories at each of the three universities. The population from which the sample was therefore all listed students in first and final semesters of the two study programs at the three universities, resulting in altogether 945 nursing students and 738 medical students. No power calculation was conducted to guide the sample size, rather, we sent an invite to participate to all students meeting the inclusion criteria. An e-mail and two follow-up reminders with an invitation to participate in the survey, which was estimated to take approximately 10 min to complete, was sent to all listed students’ e-mail addresses. The students were asked for written informed consent before responding to the survey. Two cinema tickets were drawn out randomly to one survey participant in each semester as compensation. At the end of the survey, students were also asked to indicate if they would like to participate in a focus group discussion (FGD), and if so, to provide their e-mail address. All students who had provided their e-mail address were thereafter contacted to schedule a time for a FGD via zoom. However, due to the difficulty of recruiting students at time points that suited several of them, individual interviews were conducted with those students where an FGD was not feasible. All FGD and interview participants we asked for verbal informed consent, which was audio recorded prior to participation in the interview or FGD.

### Data collection

The survey was designed to capture whether students had obtained education for sustainable development in their study program and whether the education was perceived as relevant and sufficient. The SDGs were used as a framework for designing the survey, and for priming the survey respondents about what is meant by “sustainable development” for the purposes of the study. Also, demographic data about sex, age, study program, semester and university were collected. The survey was collaboratively developed by the research team, pilot tested with two medical students, and distributed via the KI Survey on-line platform which is a survey hosted on secure servers at Karolinska Institutet. The responses for each statement were given on 4-point Likert scales ranging from “agree fully” to “don’t agree at all”, with an additional “I don’t know” option for each question. The survey was developed for the purposes of the present study and an English translation can be found in Appendix 1. Anonymized survey results were downloaded from the survey program as excel sheets which were then converted to SPSS for analysis.

The qualitative interview guideline was developed by the research team, including experts in qualitative research, and aimed to capture information about student experiences of their sustainable education (see interview guide in appendix 1). The study design was sequential exploratory mixed methods, implying that the qualitative interview/FGD participants were selected from among the survey participants, and the interviews/FGDs aimed to provide an in-depth understanding of the quantitative findings. The interviews/FGDs focused on overarching questions, which were further explored with relevant probes. FGDs and interviews were conducted in Swedish by the first author of the study. In preparation for the interviews, the students were sent a link to the UN SDG’s page [2] and asked to read through the overarching information about each SDG. They were also given the Swedish education Act statement of how universities should include sustainable development [18]. This was to ensure the students had a common understanding of what was meant by education for sustainable development in the interviews. The interviews and focus groups were conducted by the first author who is a public health researcher working with educational development for sustainable development at one of the universities where the study was conducted. The interviewer thus had a broad and in-depth understanding of the relevance of the SDG:s for health care professions education, but had no previous contact with the students being interviewed. While the interviewer herself endorsed an expectation that education for sustainable development is an important endeavor for medical universities, she was aware of this possible bias during the interviews and allowed and encouraged the entire range of possible student views on this matter as equally important and relevant.

The interviews were audio recorded and transcribed verbatim, and the transcribed text was converted to excel worksheets for analysis. Only the quotes that were chosen for the results section were translated to English by a bilingual researcher.

### Analysis

Descriptive statistics were used to summarize baseline data of the participants and to assess whether there were differences in the agreement levels of each survey statement between programs (medical/nursing) and universities (KI, UU, UMU). One way ANOVA was conducted, where the response mean value (omitting the “don’t know” response option) was the dependent variable and the university semester was the independent variable. All statistical analyses were performed with IBM SPSS Statistics, version 28.0.1.1 [13]. The quantitative analysis was conducted separate from the qualitative analysis, i.e. the data sets were not merged for analyses, but the qualitative results were rather used to provide in-depth understanding of the overall quantitative findings.

The interviews and FGDs were analyzed with qualitative content analysis with an inductive approach where the sub-categories, categories and themes were allowed to emerge from the data [20, 21]. The analysis was conducted by the first author of the study and was validated by the last author and any discrepancies between their interpretations of the data were discussed and resolved. In the analysis, first, any differences in findings between the three universities, the two study programs and the semesters were sought. However, since no obvious differences in findings from the three universities emerged, the qualitative analysis was conducted on all interviews and the FGDs from all the sites merged. Only findings from study semesters (first vs last semester) and education programs (nursing vs medical students) were separated, whenever findings differed between them.

## Results

Altogether, *N* = 294 students (18%) participated in the survey, and the breakdown of the recruitment variables into universities, programs and semesters are shown in Table 1. The mean age of the participants from all programs was 24 years. Participants from the nursing program were 92% were females and 7% were males, while 0,7% indicated neither sex. Among medical program participants, 64% were females and 33% were males, while 3% indicated neither sex.

**Table 1:** Table of survey participants

Gender N (%)	Female 226 (77); Male 62 (21); Other 4 (1,4); Prefer not to say 2 (0,7)											
Age range (mean)	18–49 (25,6)											
Study program	Medicine						Nursing					
N (%)	157 (53)						137 (47)					
Semester	First			Final			First			Final		
N (%)	86 (55)			71 (45)			85 (62)			52 (38)		
University	KI	UU	UMU	KI	UU	UMU	KI	UU	UMU	KI	UU	UMU
N (%)	41 (48)	25 (29)	20 (23)	24 (34)	20 (28)	27 (38)	31 (37)	34 (40)	20 (24)	22 (42)	10 (19)	20 (39)

**Fig. 1:**
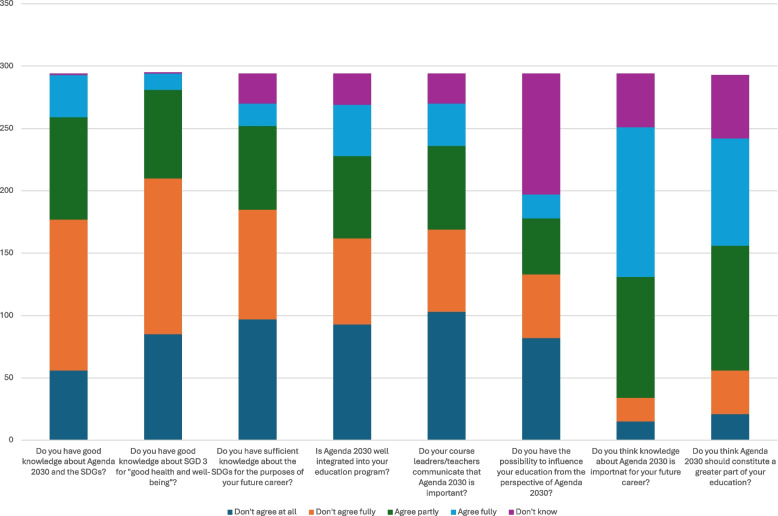
Survey responses per question and response category

Figure 1 presents the responses from the entire participant group, broken down into the different survey questions and response categories. For the first survey question, “do you have good knowledge about Agenda 2030 and the SDGs?”, the majority of students responded either “don’t agree at all” (19%) or “don’t agree fully” (41%). For the second question “do you have good knowledge about SGD 3?”, also the majority responded “don’t agree at all” (29%) or “don’t agree fully” (43%). In contrast, for the 7th survey question”do you think knowledge about Agenda 2030 is important for your future career?”, the vast majority of students responded either “agree partly” (33%) or “agree fully” (41%). This was also true for the eighth question, “do you think Agenda 2030 should constitute a greater part of your education?”, where 34% responded “agree partly” and 29% responded “agree fully”.

There were no significant differences between study programs (medical or nursing) in the reported levels of knowledge about the SDGs and Agenda 2030 overall or SDG3 in particular, nor in the self-assessed knowledge about the SDGs for the purposes of their future careers. Nursing and medical students did not differ significantly in their responses regarding whether Agenda 2030 was sufficiently integrated into their program, their ability to influence their program content in relation to the SDGs and Agenda 2030, the perceived importance of the SDGs for their future career or reporting that there should be more education on the SDGs in their programs. However, medical students were more likely to report that their course leaders/teachers had emphasized the importance of the SDGs for their education than for their future career (*p* = 0.004).

There were no significant differences depending on whether students were in first or last semester regarding the reported levels of knowledge about the SDGs overall, or SDG3 in particular, nor in the perception of having sufficient knowledge about the SDGs. However, students in their first semester were more likely to report that education about the SDGs was included in their programs, compared to the students who were in their final semester (*p* = 0.017). Moreover, the communicated importance of the SDGs by course leaders/teachers, the perceived ability to impact the content of their education, or the perceived importance of being educated in the SDGs did not differ depending on how far the students had progressed in their programs.

There were differences between the universities that related to knowledge about SDGs, where students from UU reported higher levels of knowledge of SDG3 than students from UMU (*p* = 0.038). Students from KI were more likely than students from UU or UMU to report having sufficient knowledge about the SDGs for the purposes of their future career, and students from UU were also more likely to report higher levels of adequate knowledge about the SDGs for the purposes of their future career than UMU students (*p* = 0.002). Also, the same pattern of differences between the three universities emerged in the perception of the communication for course leaders/teachers about the importance of the SDGs (*p* = 0.012). On the contrary, regarding the perceived ability to influence their education about SDGs, the perceived importance of the SDGs for their future career and the opinion that there should be more education about the SDGs in their study programs, students from UU agreed most to the statements, whereas KI students agreed second most and UMU students agreed least, with significant differences (*p* = 0.012); (*p* = 0.31); (*p* = 0.002).

Altogether 21 students participated in in the qualitative part of the study; FGDs (12 students) and individual interviews (9 students) – see Table 2. These students did not clearly differ from the overall survey participants in their responses to the survey: A majority of the qualitative study participants (52%) responded either “don’t agree at all” or “don’t agree fully” to the first survey question, “do you have good knowledge about Agenda 2030 and the SDGs?”; the majority (71%) also responded “don’t agree at all” or “don’t agree fully” to the second survey question “do you have good knowledge about SGD 3?”; and the majority (76%) responded “agree partly” or “agree fully” to the eighth question, “do you think Agenda 2030 should constitute a greater part of your education?”. However, they differed from the survey responders in their response pattern to survey question 7”do you think knowledge about Agenda 2030 is important for your future career?”, with the most common answer (28%) being “I don’t know”.

**Table 2:** Sociodemographic background of participants in qualitative study, and numbers of participants divided by study program and university

Sex/gender		19 females, 2 males											
Age range/mean		19–37/25,8 years											
University		KI				UMU				UU			
Study program		Nurse		Med		Nurse		Med		Nurse		Med	
Semester		First	Final	First	Final	First	Final	First	Final	First	Final	First	Final
N interview	9	1	-	-	2	1	1	-	-	-	2	-	2
N FGD	12		2	4				2		2		2	

*KI* Karolinska Institutet, *UMU* Umeå University, *UU* Uppsala University, *FGD* Focus Group Discussion

**Fig. 2:**
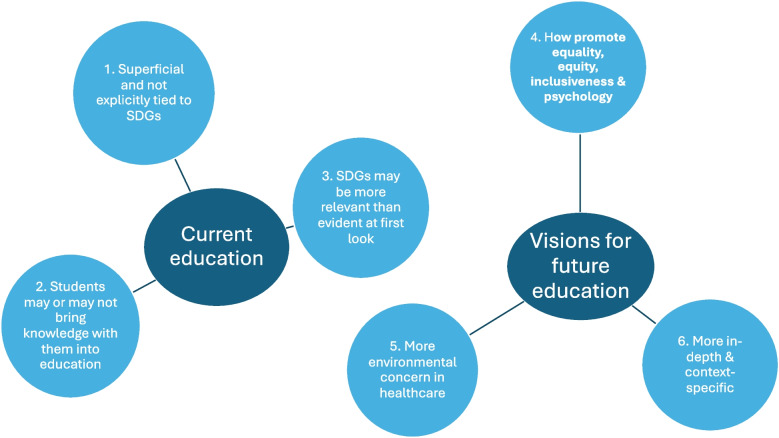
The themes and categories that emerged form qualitative analysis

The length of the interviews varied between 20 and 30 min, while the FGDs lasted between 45 and 60 min. The analyses of this data allowed for an in-depth understanding of why students in the survey witnessed to not having sufficient knowledge about the relevance of the SDGs for the purposes of their future profession and why Agenda 2030 should constitute a greater part in their education, as well as how teachers might effectively incorporate this knowledge into the programs. Data saturation did not guide the number of interviews/FGDs, as we included all students in the qualitative study who were willing to participate. However, during the course of the data gathering, we did experience that data saturation was achieved. The analysis resulted in two themes: 1) “current education” and 2) “visions for future education”, with altogether six categories, described in the following with quotes that illustrate some of the prominent perspectives more in-depth. The themes and categories are illustrated in Fig. 2. In the text we have provided participant quotes to illustrate the sub-categories, and to denote which participant the quote came from we have indicated the participant sex, study semester, study program and university acronym (KI, UMU or UU).

### Current education

The overarching theme “current education” consisted of the following categories, described below.

#### Superficial and non-explicit education on SDGs

First semester medical students mentioned that they had received some form of education about the SDGs, which ranged from the SDGs having been mentioned at some point, to a whole week addressing sustainable development. The topics thus covered included equity as well as sustainable development in health care. However, some of the medical students felt that this education had been rather superficial.

First semester nursing students also recognized that they had received education on the SDGs, for example in relation to public health work, and focusing on equality and equity in health, as well as in relation to antimicrobial resistance. However, in some instances this education was described as superficial and as having felt like "ticking a box”.

Last year medical students described that they had all received education about the SDGs, but to varying degrees: Some said that they had only been mentioned at some particular lectures such as in relation to environmental factors as risk factors for stroke, primary prevention of chronic illnesses, global child health, water-borne infections, vaccination, the effects of certain medications on the environment and social determinants of health. Others described that they had received more in-depth education on the SDGs, mainly through elective courses in for example “global health”, which they described had been inspiring. Moreover, some described that for example aspects relating to gender equality and inclusiveness in health care had been taught in depth, but not clearly tied to the SDGs.

The final year nursing students also described having received more in-depth education on the SDGs if they had chosen an elective course on "global health”. Otherwise, the SDGs had seldom been explicitly mentioned, apart from in a few instances. Some mentioned that ecological sustainability had been brought up in their clinical training, regarding sustainable material choices and the effects of medications on the environment. Some also remembered having received an introductory lecture about the SDGs in their first year of study. Equality and equity were also described by these students to have been taught about in-depth, but not related explicitly to the SDGs:

*"We may have talked about some of these topics more than the other topics. For example, issues of gender equality, they usually come up during education where they talk a lot about healthcare on equal terms, that you must give the right care to everyone regardless of what background they have or other things."* (Female, 11th sem. medical, UU)

#### Large variety in student knowledge

The first year medical and nursing students had a variety of previous knowledge about the SGD:s, for example some had previous university education where the topics of sustainable development had been brought up, while others had learnt a lot about the SDGs and sustainable development in high school.

*“My primary school education was quite good, they had a huge focus on it, at least my handicraft teacher, back then there was a huge focus on the global goals, you had to connect your own project to the global goals.”* (Male, 1st sem. medical, KI)

Others had heard about sustainable development at their workplaces as well. Further, other participants had no prior knowledge and had never heard about sustainable development prior to their medical or nursing education.

Some of the first semester nursing students described personal engagement in sustainable development issues, such as being motivated by a love for nature and animals, or thoughts about future generations’ well-being in case they were to have children of their own.

Some of the final year medical students also described having strong personal engagement in sustainable development issues, for example engagement in student associations for sustainable development as well as labor unions. Others, however described that even if they were interested to engage in these issues, they found that the education program was demanding to the extent that they didn't have much time or energy left for extra-curricular engagements.

#### SDGs more relevant than initially thought

While some did think it was important to work toward realizing the SDGs they found that it was difficult to understand how they could contribute to this in a positive manner through their medical vocation—also, the education they have received had not helped them understand this:

*"Most of all, I kind of miss the connection to the medical profession, how you yourself as a doctor can contribute to the goals. And this lecture last week, it was also like more about the bigger picture. Not about how you yourself can influence something."* (Female, 1st sem. Medical UU)

A final year nursing student also described that it was difficult to impact sustainability issues through the nursing practice:

*"In the hospital, there are certain things like how to dispose of things, throw things away, manufacture things, where you buy things from, it feels like it's sort of a management issue and not something that we nurses on the floor really have any influence on."* (Female, 6^th^sem. Nurse, KI)

However, at a closer look, some final year medical and nursing students did find that the SDGs were relevant for their clinical practice, for example if they met patients from difficult socio-economic conditions and could thus impact their psychosocial health status in various ways. Overall, some of them also emphasized the importance of medical practice taking environmental aspects into account. Yet others thought that the SDGs had no relevance for their future practice in medicine:

*"I think [sustainable development] is relevant, but I won't become a better doctor in terms of knowledge through knowing these global goals, but the way I act as a doctor, I won't act based on what the UN says, but I’ll act based on what I myself think; I want to promote equality and care on equal terms and so on."* (Male, 1st sem. Medical, KI)

### Visions for future education

The overarching theme “visions for future education” consisted of the following categories, described below.

#### Promoting equality, equity, inclusiveness and psychological aspects

Medical and nursing students in both their first and final years described various subjects related to sustainable development and the SDGs that they thought they could learn more about during medical education. One of these subjects was equality and discrimination. Some described that they found it would be of importance to know how to promote inclusive health care and not to discriminate—they wished for more practical knowledge and skills for how to provide inclusive and non-discriminatory health care. Intercultural knowledge was also called-for, in terms of an increased understanding of the needs of patients with non-Swedish cultural or language backgrounds.

*"I would still say that these intercultural meetings are important precisely because you know that these people receive lower quality care. And it is very relevant because, according to the law, everyone must be given equal care, regardless of background. But this is like a factor that you cannot influence in the same way: there are no guidelines that facilitate that meeting with those patients, or I am not aware of them.”* (Female, 6th sem. Nursing, UMU)

Some topics related to equality and equity that the nursing students thought would be relevant to learn more about included how to communicate with people from a different cultural/educational background. Moreover, some medical and nursing students expressed an interest to learn more about global health problems, as well as the illness panorama that impacts migrant populations. Some medical students wished for more knowledge and practice on how to interpret and meet a patient’s non-verbal behavior:

*"But in the medical program, I think it's important to learn about meeting people like, how to interpret a person's non-verbal signals and the like. If you're going to work with people, it feels very important that you are good with people, both from the point of being able to see people but also from the fact that you yourself are sympathetic*.*"* (Female, 1st sem. Medical UMU)

Many medical as well as nursing students expressed awareness of the stressful nature of their future work, relating to that many young clinicians burn out early in their careers. They thus hoped for more education about how to handle such emotional stressors, and wished to learn more about what organizational support structures exist that they could possibly turn to if needed.

#### Environmental awareness in health care

Both medical and nursing students mentioned various ecological sustainability issues that they would have liked to learn more about during their education programs. For example, some medical students would have liked to learn more about how pharmaceutical waste impacts the environment, so that they could make informed choices in their medical practice.

Some medical students expressed a personal interest in climate change issues, but thought it was difficult to see how these were connected to human health and wished therefore to learn more about these connections. Some expressed that health care at large seemed to lack appropriate concern for its climate impact—for example by unnecessarily using helicopters or other forms of operation that have large climate impacts. Moreover, medical as well as nursing students wished to learn more about the environmental impact of the materials they use in hospitals, such as single-use items, and to learn about more sustainable options. The impact of chemicals and pollution on health was yet another aspect that medical students wished to learn more about:

*"I've even heard that fertility is affected by emissions and chemicals and like that there will be more in the future that people will have problems with their fertility and it also feels very relevant to us as doctors. It obviously depends on the specialty, but you want to prevent or be able to remedy anything that people can have problems with."* (Female, 1st sem. Medical UMU)

#### In-depth and context-specific teaching and learning

Both medical and nursing students had various suggestions about how sustainable development and the SDGs could be taught in their study programs. Some described that they would prefer having it more in-depth in the form of a module of its own, instead of being spread out as small information points here and there throughout the education. The importance of practical examples was emphasized by many.

More specifically, medical students wished for interactive forms of pedagogy to learn about sustainable development, such as group work, work shops and panel debates. Regarding time placements, these topics were suggested to be taught after exams, as those periods were less stressful.

Yet others – both nursing and medical students – would be interested to learn about the connections to sustainable development and the SDGs throughout their education period, when relevant connections can be made. More specifically, courses in which SDGs could be brought up were suggested, for example courses in professional development towards the end of the program:

*"Because it is so important and I think that since we live in Sweden and we should be such a well-developed high-income country and then I think we have obligations."* (Female, 6th sem. Nursing, KI)

Moreover, several nursing students mentioned that the bachelor’s thesis work would have been a good place for the integration of the SDGs into their education. One nursing student problematized learning too much about sustainable development, if not given practical examples of how to contribute to it through their clinical work:

*"The global goals would feel too far away if you kept saying "and this is what the UN, those over there in New York, have talked about and decided that we should do with the whole world; you will make sure that it becomes equal and healthy and that the gaps are reduced through your work”, while like global political decision-makers don’t. Yes, but that's just not an attitude that you go into the [nursing] program with, I think. If you sort of formulate it that way then you feel that "oh now it's up to me to solve this". It becomes a bit more manageable when you can kind of say "okay this is what I'm going to do. I'm going to treat my patients this way or I'm going to make sure that I'm not causing healthcare-associated infections and stuff like that” instead of like, 'I'm going to solve the cholera problem worldwide or something'. It's kind of too big."* (Female, 1st sem. Nursing UU)

While on the other hand, learning about the SDGs more in terms of giving a broader context to their work, was thought of as positive by another nursing student:

*“I thought it would be nice to have a lecture where they kind of just talked about the global goals and why it's important that we keep them in mind. It would be nice if we got a little more context around what we do. Context around how everything we do is affected by the whole world and *vice versa* as well.”* (Female, 6th sem. Nursing, UU)

## Discussion

The findings from this suggest that a majority of medical and nursing students in the three Swedish universities did not perceive that they had learned enough about the SDGs and Agenda 2030 in their program. Further they did not feel that they had sufficient knowledge for their future career, and they would like that the Agenda 2030 and the SDGs should be a greater part of their education.

The qualitative findings formed two themes with altogether six categories and provided a more in-depth understanding. The first theme concerned the student views on the education they have received, where the category *“overall, the education that is in place is superficial or not explicitly tied to the SDGs* “ revealed that students, though having heard about the SDGs, did not perceive that they had gained any significant in-depth knowledge about the relevance of the SDGs for their future careers in health care. Indeed, similar findings have been made in other studies and settings, where students witness to superficial education for sustainable development [12, 22]. Students in our study often experienced the education that had been provided by their programs as feeling superficial and more filling the purpose of educators needing to “tick the box”.

The category *“Students have a large variety of background knowledge when entering health professional education* “, revealed that many of the students had learned about the SDGs in their prior education or workplaces, and some of them were personally engaged in activities related to promoting sustainable development. This is in line with other findings describing that students may indeed have more knowledge and be more engaged in education for sustainable development than their educators are [23], and that this is a challenge as well as an opportunity for education for sustainable development keeping up-to-date and being relevant for students [23].

The category *“the SDGs are more relevant for health care practice than what students initially thought”* was brought up by those students who described having thought more about the relevance of the SDGs for their professional practice. For example, the use of resources in health care such as transportation, the use of medications and encountering socio-economically disadvantaged groups, were ways in which our participants described that health care indeed carries an impact on sustainable development. Indeed, the main climate impact of health care is indeed incurred by procurement, transport, and medication use [24]. Moreover, a greater focus on preventive health interventions have been found to carry great potential for reducing the environmental impact of health care [24]– a fact that our study participants seemed unaware of. In addition, the SDGs explicitly state that “all learners acquire the knowledge and skills needed to promote sustainable development, including […] through […] human rights, gender equality, promotion of a culture of peace and non-violence, global citizenship and appreciation of cultural diversity” [2]—which is in line with our study participants’ descriptions of the ways in which their work can contribute to the SDGs when encountering socio-economic disadvantage.

The second theme of the qualitative findings was concerned with the ways in which students thought their education for sustainable development could be improved. In this theme, the category *“a call for more in-depth understanding of how to promote equality, equity, inclusiveness and psychological aspects in health care”* revealed that students experienced that they lacked the necessary skills and training to be able to counter inequity in health though their professional practice. Persistent study findings in low-, middle- [25] and high-income country settings [25, 26] have ascertained the patterns of inequity in health care availability and utilization. In high-income country settings in Europe, these differences in health care seeking are found predominantly in the settings of specialist health care; socioeconomic differences are not as pronounced in seeking primary health care, but more advantaged socio-economic groups are more likely to receive specialist care [26, 27]. The reasons behind this are likely due to a combination of differences in illness prevalence, access to care for example due to privatization and unequal distribution, and care-seeking behavior independent of prevalence [27, 28]. Several authors have called for medical curricula to recognize the importance of the complex interrelated socio-ecological root causes of health, well-being, and illness [29–31]. Along the lines of what our study participants described, others have also brought forth that the root causes of many conditions have been neglected in medical education [32], which focuses mainly on siloed and medicalized approaches to health [8].

In the category, *“a call for more environmental awareness in health care “* students also called for more environmental awareness and pro-activism within health care, including a greater understanding both of the environmental impacts on the health of human beings and the impact of healthcare on the environment. These aspects have indeed been described as important interlinks between sustainable development and health [2].

In the light of the present study, focusing on health care professional education, the relevant questions to ask would thus be – what skills do future health care professionals need in order to counter inequities in health? Others have suggested that public health principles, skills and resources need to be integrated into health care education curricula, with clear competency standards to guide practice and ensure impact on public health indicators [33]. Such developments would possibly also meet the call for more in-depth and context-specific teaching and learning, expressed by present study participants in the category *“A call for more in-depth and context-specific teaching and learning”*. Some recommendations that have emerged to meet this need include the development of health care educator empathy, in order to recognize the impact of the socio-ecological determinants of health, particularly regarding inequities [29]. Such an understanding should be based on a broad knowledge of the complex links between individual health and community-level data [29]. Moreover health care professionals should acquire the skills to educate individuals on beneficial health practices such as nutrition, health promotion principles and risk reduction approaches, in addition to a narrow approach on treating symptoms [29]. Indeed, overall, siloed approaches to education have been discussed not to correspond to adequate learning objectives in education for sustainable development [1, 10].

Some participants in the present study also described lacking the knowledge about how to ensure a sustainable working life for themselves, especially with regard to anticipated high levels of stress when working in health care. The calls for more training in resilience and mental health promotion among future health care professionals are indeed many, and emphasize that the lack of such training is a blind spot in much of health care professional education. Healthcare work is associated with a high prevalence of distress, which unfortunately is beginning already during their education. Also, for “transformative learning”, which has been recently emphasized by scholars of education for sustainable development, it is essential to take into account individuals’ dynamic inner dimensions and transformation [34–36], which have only recently started to receive growing attention in education and practice [36–38]. Contributing to student awareness of interconnectedness is a vital component of transformative learning. This involves a dismantling of power structures and mechanisms at play, intersectional analyses and positionality, psychological flexibility, an increased understanding of the living world including humans as primarily systemic and relational, and a recognition of society as part of nature, and the forces of localization and the globalization [39].

## Limitations and strengths

The results of the survey study should be interpreted with caution, as the participation rate of eligible students was low (18%), and there is therefore possible selection bias [40] in which students participated – perhaps those who were more interested in the topic of sustainable development were more likely to participate. Despite multiple attempts to reach eligible students via e-mail, we were not able to secure a higher participation rate. However, for the qualitative study, we may expect to have obtained a broader and more in-depth picture of the Education for Sustainable Development that the students had obtained. Since we had students participating from both targeted education programs, and almost all study semesters, we can expect that they have been able to give a representative view of their educational programs. Also, in the case of the qualitative study, a selection bias of more interested students may not only have affected the results negatively, but rather provide the views of more interested students who therefore have perhaps given the topic of sustainable development more thought than some of their possibly less interested student colleagues.

## Conclusions

Our findings suggest that students in health professional education in three Swedish Universities may lack the knowledge needed to face present and future sustainability challenges. Our findings revealed that the SDGs may be more relevant for health care practice than what is evident at a first look. For example, the use of resources in health care such as transportation and medications and how to meet patients from traumatized or socio-economically disadvantaged groups. Also, a call was made for more in-depth understanding of how to promote equity, diversity and inclusion. Psychological skills training was also asked for. Here, students experienced that they lacked the necessary skills and training to be able to counter inequity in health in their professional practice. Finally, the participants described lacking the knowledge about how to ensure a sustainable working life for themselves, especially with regard to anticipated high levels of stress.

## Supplementary Information

Supplementary Material 1.

## Data Availability

The datasets used and/or analyzed during the current study are available from the corresponding author on reasonable request.We would like to thank the study participants for their time and engagement. Also, we would like to thank Dr. Hanna Karlsson, Prof. Knut Lönnroth and Dr. Karin Leander for their contributions to the work.
