# Supplementary material for: Nursing and medical students’ views on their knowledge related to the Sustainable Development Goals – a mixed methods study at three Swedish universities
Source: BMC Med Educ. 2025 Mar 25;25:434. doi: 10.1186/s12909-025-06991-5 (PMC11934804; doi:10.1186/s12909-025-06991-5)
Supplement: Supplementary file 1 — Supplementary Material 1. [file 12909_2025_6991_MOESM1_ESM.pdf]

Focus group/individual interview guide for students

Information

Agenda 2030 includes 17 goals and 169 sub-targets, where good health and well-being is one of the goals (Goal 3). The other goals are also linked to health in various ways. Based on this, and the Swedish Higher Education Act, we would like to understand what you as students in health care professional education think about sustainable development for the purposes of your education and future career, and if and how you would like to learn about it.

Questions:

1. **Would you say you are knowledgeable about Agenda 2030 and the SDGs?**
   Probes:
   - Where did you gain this knowledge?
   - Have you learned about them previously? Was this during your current educational program, or elsewhere? If so how and when?
2. **Do you see any links between Agenda 2030 and your educational program?**
   Probes:
   - If so, why/why not? If yes, can you describe these links?
   - Have such links been brought up/discussed during our educational program, if so, how, when, in what format?
3. **Is knowledge about Agenda 2030 and the SDGs relevant for your future career in a health care profession?**
   Probes:
   - In what ways? Why/why not?
4. **Would you like to learn about Agenda 2030 and the SDGs within your current educational program?**
   Probes:
   - If so why/why not?
   - If yes, how would you want to learn about it? What forms of teaching and learning, what aspects of sustainable development?
5. **Is there anything more you would like to add or any additional comments on the topic of Agenda 2030 and SDGs in health care professional education?**

Thank you!
